# Feeling without localizing: exploring tactile misperception in a patient with uncommon parietal left brain damage

**DOI:** 10.3389/fnhum.2023.1167489

**Published:** 2023-06-23

**Authors:** Laurence Have, François Quesque, Anne-Emmanuelle Priot, Véronique Chastres, Patrice Revol, Ludovic Delporte, Eric Chabanat, Nathalie Obadia, François Cotton, Karen T. Reilly, Yves Rossetti

**Affiliations:** ^1^CNRS, INSERM, Centre de Recherche en Neurosciences de Lyon CRNL U1028 UMR 5292, Trajectoires, F-69500, Université Claude Bernard Lyon 1, Bron, France; ^2^Institut de Recherche Biomédicale des Armées (IRBA), French Military Health Service, Brétigny-sur-Orge, France; ^3^Service de Médecine Physique et Réadaptation, Plateforme Mouvement et Handicap, Hôpital Henry Gabrielle, Hospices Civils de Lyon, Pierre-Bénite, France; ^4^Service de Radiologie, Hospices Civils de Lyon, Centre Hospitalier Lyon Sud, Pierre-Bénite, France; ^5^Creatis Lab–CNRS UMR 5220–INSERM U1206 Université de Lyon, Université Lyon 1, Lyon, France

**Keywords:** somatosensory processing, tactile localizing, visuo-tactile integration, multisensory, parietal lesion

## Abstract

**Introduction:**

We report a very unique clinical presentation of a patient who complained, after a left parietal brain damage, about feeling tactile stimulations on his right upper limb without being able to localize them.

**Methods:**

Using a single case study approach, we report three experiments relying on several custom-made tasks to explore the different levels of somatosensory information processing, ranging from somato-sensation to somato-representation.

**Results:**

Our results showed a preserved ability to localize tactile stimuli applied on the right upper limb when using pointing responses while the ability to localize was less efficient when having to name the stimulated part (akin Numbsense). When the stimuli were applied on more distal locations (i.e., on the hand and on fingers), the number of correct responses decreased significantly independently of the modality of response. Finally, when visually presented with a stimulus delivered on the hand of an examiner in synchrony with the stimulation on the hidden hand of the patient, responses were largely influenced by the visual information available. Altogether, the convergence of these different customized tasks revealed an absence of autotopagnosia for motor responses for the right upper limb, associated with altered abilities to discriminate stimulus applied on distal and restricted/closer zones in the hand.

**Discussion:**

The somato-representation of our patient seemed to significantly rely on visual information, leading to striking deficits to localize tactile stimuli when vision and somesthesic afferences are discordant. This case report offers a clinical illustration of pathological imbalance between vision and somesthesia. Implications of these troubles in somato-representation on higher cognitive level processes are discussed.

## Introduction

There is a long tradition in neuropsychology of attempting to link brain lesions to specific pathological presentations. Among the most established phenomena, left-right functional asymmetry has been identified by clinical manifestations attributed to unilateral brain damage (e.g., aphasia, hemispatial neglect, and apraxia). In general, body perception disorders are present when the right hemisphere is damaged (Semenza and Goodglass, 1985; Coslett, 1998; Palermo et al., 2014; Rousseaux et al., 2014) while lesions in the left hemisphere have been associated with language disorders, affecting either its understanding (e.g., Wernicke’s aphasia), its production (e.g., Broca’s aphasia). Left-sided lesions are also associated with apraxia (Goldenberg, 2009, 2013; Goldenberg and Randerath, 2015; Etcharry-Bouyx et al., 2017). However, in some cases, basagnibody perception can also be affected after left brain damage, especially after a lesion of the parietal lobe. As a typical example, the Gerstmann syndrome is also characterized by finger and toe agnosia and left-right confusion which might evoke body perception disorders (Strub and Geschwind, 1974; Mayer et al., 1999; Vallar, 2007; Rusconi, 2018; Basagni et al., 2021). It remains unclear, however, to what extent the latter clinical manifestations are related to language access (e.g., knowing the name of the fingers) or to body representation (e.g., not identifying the fingers).

Historically, body perception disorders were roughly attributed to the parietal lobe on the basis of clinical observations, but more recent works allowed a precise description of the cerebral substrates associated with different kinds of body awareness disorders (Pisella et al., 2019). For example, the right temporo-parietal junction is implicated in autoscopy (i.e., “the perception of one’s own body either from an internal point of view, as in a mirror or from an external point of view,” Anzellotti et al., 2011) and out-of-body experiences (i.e., “the perception of one’s own body and of the world from a location outside the physical body,” Park and Blanke, 2019; Quesque and Brass, 2019). Different psychological constructs related to various levels of consciousness might actually be covered by the term body perception.

Historically, body schema (e.g., implicitly considering the physical limits of our body for action) was opposed to body image (e.g., the conscious image of our own body, Paillard et al., 1983; Gallagher, 1986; Longo and Haggard, 2012). The notions of body schema and body image were introduced a century ago by Head and Holmes (1911). Body schema refers to low-level sensorimotor processing involved in perceptual coding and motor reactions. The body schema is multimodal and requires implicit knowledge of body position in space, as well as information relative to the size and shape of body parts (Rossetti et al., 1995, 2001; Cardinali et al., 2009; De Vignemont, 2010), and allows us to interact efficiently in our environment. In contrast, body image is often defined as a conscious representation of one’s own body (Head and Holmes, 1911; Paillard et al., 1983). It is influenced by psychological considerations, such as the emotional state, mood and self-esteem (Blanke, 2012). Thus, body representations can be affected by biased body schema, body image, or their interactions.

In order to precisely categorize altered body representations, Longo et al. (2010) proposed a more fine-grained classification in which three different dimensions are distinguished. The first dimension, somato-sensation, corresponds to primary sensory processing. The second dimension, somato-perception corresponds to the localization of somatic and postural stimuli. The third dimension, somato-representation, corresponds to general semantic knowledge and body consciousness about the organization and metric properties of body parts, and the schematic representation of their functional relationship. If the first and third dimensions can be linked to body schema and body image, the second dimension (somato-perception) could represent an intermediary level between these two psychological constructs.

Clinical practice requires a precise understanding of the interactions between the different dimensions of body representations. Identifying disturbed cognitive processes is primordial for guiding clinical decisions and the provision of appropriate and efficient rehabilitation to patients. An extraordinary patient admitted in our rehabilitation department confronted us with the importance of understanding the various dimensions of body representations. The patient suffered from a post-surgical haemorrhagic injury in the left parietal lobe and the conclusions of a somatosensory perception assessment performed during a routine clinical exam were that there was no sensory loss. Indeed, the patient correctly detected every tactile stimulus applied to his right hemi-body. However, at the end of this tactile detection test, he expressed clear astonishment and reported that “[he] felt all the stimuli, but had absolutely no idea which part of his body was stimulated!” This type of difficulty could reflect an alteration in any of the three dimensions described above, but to date, no routine examination has been available to precisely categorize this type of deficit.

The goal of the present study was to present a description of the patient’s alteration in body representation and to assess it at different perceptual levels. We developed an exploratory strategy based on experimental contributions which can be used in clinical settings. In a set of three experiments, we assessed the abilities of our patient to localize tactile stimuli focusing on (1) the influence of the localization of the stimulated parts (proximal, distal), (2) the influence of the modality of responses (self-directed pointing, pointing on a drawing, naming), (3) and the contribution of visual input (no visual input, visual interferences).

## Case report

**FIGURE 1:**
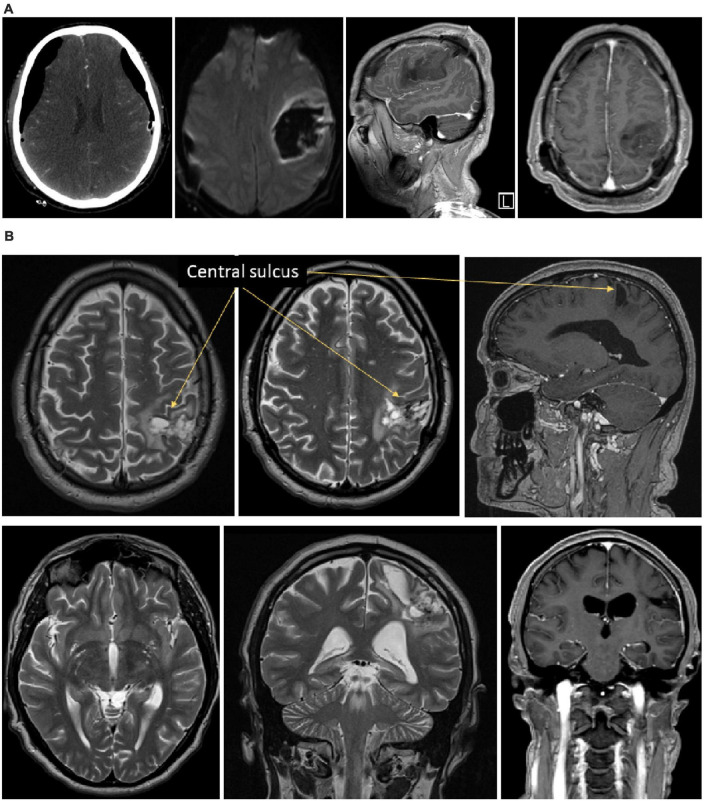
Illustration of the brain lesion of Mr. I. Panel **(A)** Onset CT-scan of Mr. I’s brain revealing acute hematoma with mass effect affecting frontal and parietal lobes, pre- and post-central gyrus. The pyramidal pathway was affected to a lesser extent. Panel **(B)** 6 months post-onset MRI showing a remaining porencephalic cavity in a post-central location, affecting the superior parietal lobe, the posterior part of the para-central gyrus, the anterior part of the supra-marginal gyrus. A portion of the arcuate fasciculus is affected too. No remaining mass effect is observed.

We report the case of a 42-year-old male patient (Mr. I), who presented with a left acute hématoma, affected frontal and parietal lobes, pre et post-central gyrus, and the pyramidal pathway to a lesser extent (see Figure 1) due to surgical treatment of a subdural hematoma 3 years earlier. In the first 3 weeks after the injury, he presented a right hemiplegia and right multimodal sensory loss. Initially, oral and written expression, articulation, calculation, and writing were impaired. In the acute period, rehabilitation focused on the motor deficit of the right, dominant, hand and speech therapy. The motor deficit partially resolved within less than 2 months and Mr. I appeared to have recovered normal somaesthetic perception. Then, rehabilitation was continued regularly to improve motor control of the right upper limb. Oral and written expression, and articulation deficits were resolved at the time of our investigation. During one of the rehabilitation sessions, after a 6 month-follow up period, Mr. I described an atypical and striking situation. He was sitting with his 7 year-old son on his legs and while his son started to move his right upper limb to catch a toy on the nearby table, Mr. I experienced ownership of his son’s arm. He explained that he had the very uncomfortable feeling that the right upper limb of his son he was seeing in a self-referential was his own limb. The feeling of ownership was present despite the anatomical incongruence: the arm and the hand of his 7 years old son were much shorter than his own; obviously, the dimensions and proportions were not the same as his own upper limb, and were incongruent with his healthy side. This experience was all the more disturbing for him that the feeling of ownership was not associated with the feeling of agency. Mr. I described the feeling of the right upper limb of his son as his own, but not the control motor of it, which led to the very uncomfortable experience that his arm moved on its own. The patient was perfectly aware of the incongruence of this feeling and therefore was very stunned by this experience. He aspired actively to understand and seeked scientific explanations for this peculiar experience. He aspired actively to understand and to obtain scientific explanations for this experience.

Finally, it is crucial to note that our patient was a medical doctor and consequently had a high level of knowledge about physiology, neurology and psychiatry. He was puzzled by the phenomenon and consulted us in search of explanations.

During the following rehabilitation sessions, Mr. I chronically complained about the feeling of having “three right upper limbs” appearing when his right upper limb was not in his visual field. When a tactile stimulus was applied to his right upper limb while blindfolded, he reported that he experienced “a limb on which [he] perceived the tactile stimulus,” “a limb that [he] was able to move” and a third limb “in a felt position and localization.” According to him, none of the positions of these three right upper limbs were spatially congruent. Over time, he observed that these manifestations were reproducible in many situations of his daily life, such as driving, playing with his children or lying in his bed. This phenomenon led to difficulties shaking hands or grasping objects and led him to systematically look at his right hand while moving it. As a strategy to avoid or reduce the feeling of having non superimposed multiple right upper limbs, the patient kept his right hand in contact with his stomach, limiting any spontaneous mobility.

Following his complaints, a routine clinical and paraclinical evaluation of sensory perception was conducted. No pallesthesic deficit was found. Thermal and algesic stimuli were perceived in both upper limbs. Sensations were, however, exacerbated in the right upper limb, suggesting some hyperesthesia. Tactile discrimination (2-point discrimination test) and perception threshold (Von Frey monofilaments) were normal. Under visual control, no body-anomia or autotopoagnosia were noticed. In contrast, the localization of tactile stimulation was altered in the right upper limb but preserved in the left upper limb.

Clinical examination was completed by control MRI and electrophysiological examinations. The MRI performed 1 year after the onset showed no mass effect anymore, but a remaining porencephalic cavity, in a post-central location, affecting the superior parietal lobe, the posterior part of the para-central gyrus, the anterior part of the supra-marginal gyrus. A part of the arcuate fasciculus is affected too (see Figure 1). Somatosensory evoked potentials and laser evoked potentials were performed to explore the somatosensory pathways and the pain pathways, respectively. Somatosensory evoked potentials revealed no N20 response in the left primary somatosensory cortex S1 N20. The P27, P45 and N60 were also absent after stimulation of the right upper limb. Only a very weak, non-lateralized, N30 component was detectable. Overall, this pattern of results revealed a severe disturbance of the left parietal cortex. Laser evoked potentials showed wider cortical responses when the stimuli were applied to the right hemi-body than to the left hemi-body. This is consistent with the hyperesthesia clinically observed on the right upper limb.

**FIGURE 2:**
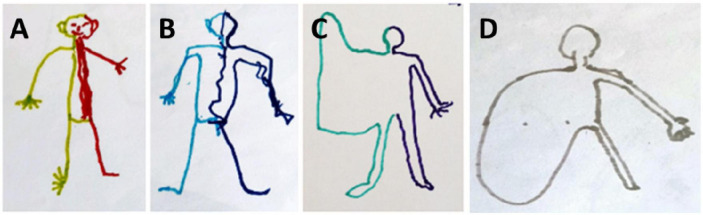
Evolution of the drawing-a-man test during 3 years of follow up. **(A)** First test 4 months after the brain injury. **(B)** 2nd test at 12 months. **(C)** Third test at 24 months. **(D)** Fourth test at 36 months.

During the 3 years of follow up, our patient was asked to draw a man to illustrate how he represented his body. These drawings considerably evolved through time as can be seen in Figure 2. In the first drawing, two different colors were spontaneously used to represent the two hemi-bodies. Interestingly, his extremities (i.e., fingers and toes) were depicted. Then, the toes disappeared. The main change in the drawing over time was the disappearance of the right upper limb, and the representation of the upper limb by a large area no longer separated from the trunk. The following drawing showed the disappearance of the lower limb and the right hemi-body represented by a large area with no separation between the upper limb, the lower limb, and the trunk.

## Overview

The patient has been followed up for 3 years, since the beginning of the rehabilitation procedure, i.e., 4 months after the lesion. In order to characterize the level of the body representation disorder in the very unique clinical presentation of Mr. I, we investigated tactile perception using several custom-made tasks. Thus, three experiments were performed 3 years after the onset, while the clinical presentation was non-evolutionary anymore, to explore the different dimensions of somatosensory information processing, ranging from somato-sensation to somato-representation. The sequence of experiments was defined so as to respect a logical progression from a lower cognitive level of body perception to higher cognitive level of body representation.

In all three experiments, Mr. I was sitting on a chair, with his right hand and forearm resting on a table. The palmar face of the hand was in contact with the table and the back of the hand was exposed to the tactile stimulations. Mr. I’s participation was facilitated by the fact that he was a medical doctor and had a scientific and neurological background. He was very enthusiastic about engaging in the various experiments and was motivated to better understand his clinical manifestations. Nevertheless, on specific occasions he was emotionally and cognitively perturbed by his perception, especially when he perceived a discordance between visual and tactile information. On a few occasions, when a stimulus was delivered, he exclaimed “it’s disgusting!”, or “it’s not possible that you touched my hand right there!” Despite this very transient discomfort, he remained very curious and very patient throughout the hours of testing.

## Experiment 1

### Materials and methods

In the first experiment, the objective was to characterize tactile perception. We assessed Mr. I’s ability to detect a tactile stimulus, depending on its location on his right upper limb and on two modalities of response. Thus, tactile stimuli were delivered by an examiner using a monofilament on four different locations on the right upper limb (dorsal face of the hand, wrist, distal forearm, and proximal forearm). Six trials were delivered at each location in a totally randomized order for a total of 24 trials. For each trial Mr. I provided two responses: *pointing* (i.e., After each stimulation, Mr. I had to point with his left index to the location on which he felt the tactile stimulus on his right upper limb) and *naming* (i.e., After each stimulation, Mr. I had to name the location on which he felt the tactile stimulus on his right upper limb). Mr. I was blindfolded during the whole experiment and did not receive any feedback about his responses during the whole experiment.

### Results

In the *pointing* condition, 100% of the responses were correct (24/24) and showed no signs of hesitation when responding. In the *naming* condition, he correctly named the stimulated locations on only 62.5% of the trials (15/24). For seven of the nine trials on which he misnamed the location his response corresponded to the nearest proximal localization (e.g., for a stimulation on the hand he reported that he perceived it on his wrist). Only two erroneous responses referred to a more distal location: one to the nearest distal localization (stimulation of the proximal forearm perceived on the distal forearm) and one to a more distant distal localization (stimulation of the distal forearm perceived on the hand).

To better characterize the discriminatory sensory abilities of the patient we computed d’ (Bonnet, 1986) for each location (see Table 1). Classically, d’ is based on two values: correct positives (stimulus detected in a given location which corresponds to the actual location of the stimulation) and false positives (stimulus detected in a given location which does not correspond to the actual location of the stimulation). A d’ close to 0 reflects random responses.

**TABLE 1:** Proportion of correct, false positive responses, and d’ for the *naming* condition in experiment 1.

	Correct detections (%)	False alarms (%)	d’
Thumb	50.00	33.33	0.43
Index	0	10.00	−4
Middle finger	33.33	23.33	0..30
Ring finger	16.66	3	0.91
Little finger	50.00	7	1.5
Hand	66.66	0	5
Mean	36.11	12.77	0.78

The mean d’ (1.47) revealed a relatively preserved ability to localize tactile stimulations when the response required Mr. I to name the location, with the least precise responses obtained for the distal forearm (*d*’ = 0.79). In order to compare his performance in the *pointing* and *naming* conditions, we computed an exact Fisher test on the proportion of correct responses per location between conditions. This revealed a significant deviation in responses from the flat distribution (*p* = 0.002) with an advantage for the pointing condition. Taken together, our results show that Mr. I’s ability to point to tactile stimuli on different large parts of his upper right limb was preserved and that he also has a relatively preserved ability to explicitly identify the location of tactile stimuli. Since our paradigm did not allow us to distinguish between lexical and representational deficits in the naming condition we designed a second experiment to address this issue.

## Experiment 2

### Materials and methods

In the second experiment, the objective was to characterize tactile perception on the more distal part of the right upper limb (the hand). We assessed Mr. I’s ability to detect a tactile stimulus, depending on its location on his right upper limb and on three modalities of response. Compared to experiment 1, the stimulated zones were restrained and the addition of the modality “pointing on a drawing of the hand” allowed to assess a higher cognitive level of body representation. Tactile stimuli were sequentially delivered by an examiner to six locations (distal phalanx of each finger and the center of the dorsal face of the hand) on Mr. I’s right hand. As in Experiment 1, stimuli were a monofilament and the order of the locations was randomized, with six trials per location for a total of 36 trials. In contrast to Experiment 1, in this experiment three response conditions were used: *naming* (as in Experiment 1), *pointing* (as in Experiment 1), and *pointing on a drawing* representation of his hand. Mr. I was blindfolded during the *pointing* and *naming* conditions and his hand was out of sight during the *pointing on a drawing* condition. The patient did not receive any feedback on his responses. This task, and in particular the *naming* condition, was judged to be difficult by Mr. I, who often reported not being able to respond confidently. When he was unable to provide an answer, we encouraged him to respond as in a forced-choice paradigm by proposing the names of the different locations.

As in Experiment 1, we computed d’ for each location in the *naming* condition. Moreover, the proportion of correct responses were compared across locations through a Pearson chi square test. In the *pointing* and *pointing on drawing* conditions, we computed the mean x and y pointing coordinates for each location and its standard deviation in x and y. The x and y distance errors were computed from the x and y vectors between the mean coordinates of each pointing location and the stimulated location. Mean normalized variability associated with these two vectors was also computed for each stimulated location. For each dimension, statistical analysis was performed on the mean distance errors using a Locations (Thumb, Index finger, Middle finger, Ring finger, Little finger, Hand) × Conditions (pointing, pointing on drawing) analysis of variances with repeated measures on Conditions. In case of significant results at the Levene’s test (i.e., homogeneity test), non-parametric alternatives (Kruskal–Wallis ANOVA) were conducted. Post hoc comparisons were conducted using *t*-tests with Bonferroni correction.

### Results

#### Identification

**FIGURE 3:**
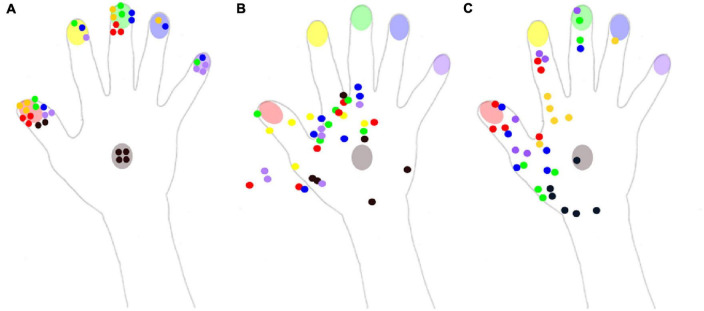
Reported localization of the tactile stimuli for the three conditions of experiment 2. The color of each dot corresponds to the color of the stimulated part (red: thumb, yellow: index, green: middle finger, blue: ring finger, purple: little finger, black: hand). **(A)** In the naming condition, Mr. I had to name the localization on which he felt the tactile stimulus on his right hand. **(B)** In the pointing condition, Mr. I had to point with his left index the localization on which he felt the tactile stimulus on his right hand. **(C)** In the pointing on a drawing condition, Mr. I had to point with his left index the localization on which he felt the tactile stimulus on a drawing of his right hand.

In the *naming* condition, out of the 36 stimuli delivered on the right hand, only 13 were correctly identified. A response bias was observed in favor of the thumb and the middle finger (respectively named 13 and 9 times out of the 36 trials), as can be seen in Figure 3A. The mean d’ (0.78, see Table 2) revealed an altered ability to localize tactile stimuli on the right hand, with the least precise responses obtained for the middle finger (*d*’ = 0.3). Interestingly, the mean d’ obtained in Experiment 2 was smaller than that obtained in Experiment 1, when stimuli were applied to the various locations on the upper limb.

**TABLE 2:** Proportion of correct, false positive responses, and d’ for the *naming* condition in experiment 2.

	Congruent	Non-congruent	Control
Fingers	100% (9.8)	0% (8.9)	0% (4.3)
Hand	91.66% (8)	66.66% (5.8)	83.33% (5.8)
Forearm	91.66% (6.8)	33.33% (5.9)	58.33% (7)

Even if the two other conditions were not directly comparable due to the non-binary nature of pointing responses, a qualitative description was still informative. In the *pointing* condition, responses were concentrated on the radial side of the hand, and numerous responses were not even on the hand, but instead were in the space next to the hand (Figure 3B). Mr. I correctly pointed to the stimulated body part on only 6 of the 36 trials. These 6 correct pointings corresponded to the 6 stimuli delivered on the back of the hand for which the high variability in pointing localizations might have been compensated by the wider surface of this zone. Overall, the present pattern of responses revealed that his performance fell below chance level for the stimuli displayed on fingers (0/30), compatible with a response bias away from the fingers. On the contrary, in the *pointing on the drawing* condition (Figure 3C), 35 stimuli were localized within the medial part of the hand, only one was localized in the ring finger and none were localized on the little finger. In addition, among the 30 stimuli applied to the fingers, only 14 were localized to a finger, revealing a strong response bias in favor of the back of the hand. None of the 12 stimuli applied to the ring and little fingers were correctly identified. Mr. I correctly localized the six stimuli delivered to the back of the hand, but five out of the six stimuli were localized to a more proximal localization. Finally, out of the six stimuli applied to the index, only two were correctly localized near the stimulated zone, while four were localized in the metacarpophalangeal joint of the index (i.e., on a more proximal location). The general spatial bias toward the 1st interosseous of the right hand in the pointing on drawing condition is clearly visible on Figure 3C (yellow dots).

#### Pointing errors in x

**FIGURE 4:**
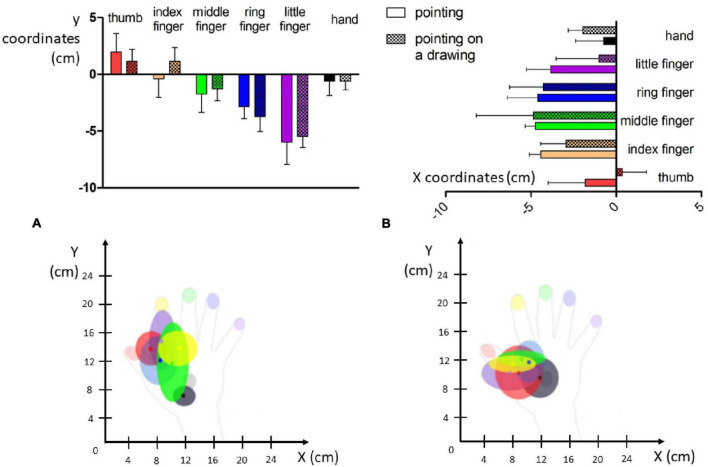
Visual representation of pointing errors. (Top) Mean distance error for each localization in centimeters (whiskers represent standard deviation). (Bottom) Mean dispersion of the pointing errors in the x and y dimensions in the pointing panel **(A)** and pointing on drawing panel **(B)**.

As illustrated in Figure 4 (Left top panel), distance errors in x did not differ significantly between the *pointing* and *pointing on drawing* conditions [*F*(1,30) = 0.215, *p* = 0.65]. Distance errors on x were, however, largely influenced by the stimulated location [*F*(5,30) = 43.03, *p* < 0.001]. Specifically, distance errors on x were highest for the little finger (significantly different from all other locations, see Annex 1), followed by the ring finger (significantly different from all other locations except the middle finger), and the middle finger (significantly different from all other locations except the ring finger and the back of the hand). There was no interaction between Conditions and Locations [*F*(5,30) = 1.56, *p* = 0.20]. Overall, analysis of the distance errors in x revealed larger errors for the most laterally stimulated locations.

#### Pointing errors in y

Regarding the distance errors in y (Figure 4, right top panel), they did not differ significantly between the *pointing* and *pointing on drawing* conditions [*F*(1,30) = 3.6, *p* = 0.07]. Distance errors in y were, however, largely influenced by stimulation location [*F*(5,30) = 13.41, *p* < 0.001]. Specifically, distance errors on y were greatest for the middle finger (significantly different from the ring finger, the thumb and the back of the hand, see Annex 1), followed by the ring finger (significantly different from the little finger, the thumb and the back of the hand), the index (significantly different from the thumb and the back of the hand). There was no interaction between Conditions and Locations [*F*(5,30) = 1.69, *p* = 0.17]. Overall, analysis of distance errors in y revealed larger errors for the most distal stimulated locations.

Taken altogether, our results reveal that when investigating hand-centered tactile perception, stimuli were mostly experienced in a restricted zone at the most proximal and radial part of Mr. I’s right hand (see Figure 4, bottom panel). In Experiments 1 and 2, Mr. I was blindfolded in order to put him in the ideal experimental conditions for investigating his tactile perception abilities. Interestingly, in real life settings, Mr. I reports experiencing ownership of other people’s arms. Thus, we designed a third experiment to investigate the impact of visual information on his tactile perception abilities.

## Experiment 3

### Materials and methods

The objective of experiment 3 was to explore the influence of visual contributions in tactile perception in Mr. I. Experiment 3 was roughly based on the rubber hand illusion paradigm (e.g., Botvinick and Cohen, 1998). Mr. I’s forearm and hand rested on a table and were covered by an opaque surface. He thus had no vision of his forearm or hand. The arm of another person of similar age and gender was placed on the opaque surface in approximately the same location as Mr. I’s arm. Tactile stimuli were applied in three different conditions. In the *Congruent* condition, tactile stimuli were applied to identical on corresponding locations on the patient’s and experimenter’s upper limbs. In the *Incongruent* condition, stimuli were applied to different locations, and in the *Control* condition tactile stimuli were applied only on the patient’s arm and not on the experimenter’s arm (i.e., no visual information regarding the stimulated location localizations was available in this condition). Mr. I’s ability to localize tactile stimuli in the presence of congruent, incongruent, or no visual information was tested in three blocks in three different parts of the upper limb (fingers-centered, hand-centered, forearm-centered). In each case the distance between the two tested body parts was the same: index finger versus ring finger; back of the hand versus wrist; proximal versus distal forearm. On each trial, Mr. I’s task was to identify where he had been stimulated by choosing between the two proposed locations. The order of conditions within each block was fully randomized and the tactile stimulation procedure was similar to that used in Experiments 1 and 2. A total of 108 trials were performed, with 36 trials per block (12 per condition). After each trial, Mr. I’s level of confidence in his response was measured by verbal response on a scale ranging from 0 (not confident at all) to 10 (extremely confident). In order to compare his performance in the different conditions, we computed an exact Fisher test on the proportion of correct responses per location between conditions. Additional two-by-two comparisons were conducted using *Z* = $\frac{P\,1-P\,2}{\sqrt{\left(P\times \left(1-P\right)\times \left(\frac{1}{n\,1}+\frac{1}{n\,2}\right)\right)}}$, with P1 and P2 corresponding to respective probabilities of correct identification for the compared conditions, with P denoting the mean probability of correct identification, and with n1 and n2 denoting the respective total sample size of the compared conditions (Rosenberger, 2017). Concerning the level of confidence, statistical analysis was performed using a Locations (fingers-centered, hand-centered, forearm-centered) × Conditions (Incongruent, Congruent, Control) analysis of variances with repeated measures on Conditions. Post hoc comparisons were conducted using *t*-tests. A Bonferroni correction was applied to all two-by-two comparisons.

### Results

#### Localization abilities

The exact Fisher test revealed a significant deviation in responses from the flat distribution (*p* < 0.01) revealing variations in the probability of correct identification. Two-by-two comparisons revealed that the proportion of correct identification was higher in the Congruent condition than in the Incongruent (*Z* = 5.4, *p* < 0.001) and control (*Z* = 4.4, *p* < 0.001) conditions. No difference was observed between the two latter conditions (*Z* = 1.2, uncorrected *p* = 0.15). Concerning the effect of locations, the proportion of correct identification was lower for stimulations displayed on the fingers as compared to the hand (*Z* = 4.05, *p* < 0.001) and arm (*Z* = 2.36, *p* = 0.009). No difference was observed between the two latter locations (*Z* = 1.8, uncorrected *p* = 0.04).

#### Level of confidence

We observed a main effect of experimental condition [*F*(2,62) = 17.5, *p* < 0.001] on Mr. I’s confidence in his responses, with higher confidence in the *congruent* condition than in the *non-congruent* [*t*(70) = 3.14, *p* = 0.002] and in the *control* conditions [*t*(70) = 5.91, *p* < 0.001]. Strikingly, Mr. I was more confident in his responses in the *non-congruent* than in the *control* conditions [*t*(70) = 2.77, *p* = 0.004]. No effect of location was observed on Mr. I’s confidence in his responses [*F*(2,31) = 2.23, *p* = 0.12], but an interaction between both variables [*F*(4,62) = 10.13, *p* < 0.001] was observed. Specifically, and as illustrated in Table 3, within the *congruent* condition Mr. I was more confident when having to localize fingers-centered rather than forearm-centered [*t*(22) = 3.53, *p* < 0.001] tactile stimulations. No difference was observed between fingers-centered and hand-centered and between hand-centered and forearm-centered stimulations. Within the *non-congruent condition* Mr. I was more confident when having to localize fingers-centered rather than hand-centered [*t*(22) = 3.53, *p* < 0.001] or forearm-centered [*t*(22) = 3.44, *p* = 0.001] tactile stimulations. No difference was observed between hand-centered and forearm-centered stimulations. Finally, in the *control* condition Mr. I was more confident when having to localize fingers-centered rather than forearm-centered [*t*(22) = 3.34, *p* = 0.002] tactile stimulations. Again, no difference was observed between fingers-centered and hand-centered and between hand-centered and forearm-centered stimulations.

**TABLE 3:** Proportion of correct responses and mean associated level of confidence in brackets for the three conditions and the three stimulated locations.

	Correct detections (%)	False alarms (%)	d’
Hand	50	5.55	1.59
Wrist	83.33	16.66	1.93
Distal forearm	33.33	11.11	0.79
Proximal forearm	83.33	16.66	1.93
Mean	62.50	12.50	1.47

## Discussion

This case report aimed to develop an exploratory experimental protocol that could potentially be used in a clinical setting to characterize alterations in body perception and body representation. A series of three experiments investigated the patient’s ability to localize a tactile stimulus on different parts of his upper limb. The unique clinical presentation of this patient, who has preserved tactile detection and tactile autotopagnosia, provided us with an opportunity to describe the relations between body perception and body representation.

In the first experiment, we examined the influence of stimulation location on the upper limb on the patient’s ability to localize stimuli with pointing versus naming responses. In a second experiment, we further explored the influence of response modality (self-directed pointing, pointing on a drawing, naming) when stimuli were applied only to the distal part of the upper limb (the hand and fingers). In a third experiment, we explored the contribution of visual input (visual input, no visual input, visual interference) on the patient’s ability to localize tactile stimuli and his confidence in his responses.

Overall, our protocol revealed body autotopagnosia that was not captured by the initial clinical examination which included only detection tasks. In Experiment 1, Mr. I was mostly able to correctly name and point the different parts of his upper limb when they were stimulated. The discrimination for localization in large areas of his right upper limb was preserved. The results of Experiment 2, however, show that he had difficulty localizing stimuli within a restricted area, as he was unable to successfully localize stimuli applied to his different fingers, whether it be using naming, pointing, or pointing on a drawing. The results of experiment 3 revealed that Mr. I’s responses depended heavily on visual input, as did his confidence in his responses.

The fact that Mr. I was able to correctly detect the presence of stimuli, but not always correctly localize these stimuli, suggests that he has difficulties related to autotopagnosia (i.e., the difficulty to determine the location of the stimulus). Interestingly, when the task involved identification of stimulus location among large zones on the upper limb (Experiment 1) he performed relatively well. However, when the task involved identification of stimulus location within a small zone (e.g., the hand in Experiment 2) he committed a significant number of errors. The pattern of responses in Experiment 1 and 2 revealed that Mr. I had most difficulty localizing stimuli when they were on the distal part of the upper-limb, specifically within the hand. More distal and lateral locations were associated with the more important deficits of identification and localization. The severity of autotopagnosia in Mr. I’s right upper limb was influenced by both a disto-proximal and a latero-medial gradient.

With the exception of stimuli applied to the back of the hand, all stimuli in Experiment 2 were localized to an area of space corresponding to the medio-proximal part of the right hand. One interpretation of this pattern is that the thumb and the back of the hand acted like attractors. The fact that many responses in the pointing conditions fell outside of the anatomical boundaries of his hand suggest that he might have difficulties locating the exact boundaries of his upper limb. This idea is supported by drawings of his body and their evolution across time, which reveal the progressive disappearance of the normal shape of the fingers, then of the hand, and finally of the whole upper limb.

The pointing end-points were mostly localized in the area of the first interosseous muscle, which likely reflects a contraction bias (regression to the mean of possible responses) which is frequently observed in situations of perceptual uncertainty (Mon-Williams and Tresilian, 1999). Consistent with this idea is the observation that errors in the X dimension on the ring and little fingers (furthest from the mean zone) were increased compared with index finger errors. Altogether, both qualitative and quantitative aspects reflect an autotopagnosia targeting specifically Mr. I’s right upper limb.

Our results suggest that pointing, naming, and pointing on a drawing provide access to different “cognitive levels” of body representation. Autotopagnosia can be assessed in verbal and non verbal conditions that reflect the level of consciousness of the localization of the tactile stimulus (Semenza and Goodglass, 1985). Pointing at a tactile stimulus when blindfolded provides access to unconscious body topognosia (Tsay et al., 2016) as, for example, when slapping a mosquito on the arm, which happens before consciously identifying the location of the mosquito and/or the nature of the stimulus. In contrast, naming the touched part of the body relies on access to more conscious somatic topognosia. In the present case, and based on the classification in three dimensions (somato-sensation, somato-perception and somato-representation) proposed by Longo et al. (2010), Mr. I’s somato-sensation appears to be preserved, as primary sensory processing seems accurate. Indeed, Mr. I could correctly detect a tactile stimulus applied on his right upper limb. Incorrect responses were observed when he was asked to localize stimuli, suggesting a problem in both somato-perception and somato-representation (consciousness concerning metric properties of body parts and the representation of their relationship to each other). The troubles in somato-perception manifested in Mr. I by the lack of perception of the boundaries of his right upper limb may be responsible for troubles in somato-representation such as the feeling of having multiple hands or as the experience of ownership over the experimenter’s hand. Thus, this unique case study illustrates how tactile sensory inputs affect higher levels of conscious body representations.

When we asked Mr. I to identify which part of his upper limb was touched he explained that he has to reason logically and cannot just base his response on his perceptual experience. For example, he said “since I felt it was my thumb which was touched, then the following touch had to be applied on my middle finger, because it seemed that the touch was far enough from my thumb.” During the experiments, Mr. I expressed his astonishment regarding the distance he perceived between his fingers which seemed to be too large. He notably said that “it is not possible that it was my thumb! It’s too far!” and reported that “It’s very difficult.”

As tactile input is important for constructing the subjective reference frame, it follows that erroneous mapping of the upper limb will affect interactions with the nearby environment. If tactile inputs cannot be relied upon for the construction of an accurate body representation, then one possible compensatory strategy could be to increase the weight attributed to the more reliable visual inputs. The results from Experiment 3 suggest that Mr. I adopts this type of strategy, as in conditions of non-congruence between the touched and viewed stimulus locations. His responses suggest that he “felt the tactile stimulations where he saw them.” In the presence of the experimenter’s hand, self-confidence in his responses was also affected by the congruency between the location of the two stimuli. Interestingly, strong self-confidence in estimation was not always associated with correct stimulus localization.

Historically, Schilder (1931) distinguished different types of finger identification impairments in terms of finger agnosia, finger aphasia, visual finger agnosia, apraxia of finger choice and constructive finger apraxia. Following these first steps, Mayer et al. (1999) reported a case of Gerstmann syndrome attributed to a small left posterior parietal ischemic lesion and characterized by the four usual symptoms without any signs of aphasia, apraxia, amnesia or intellectual deficit. In order to specify the impairments in sensory integration, he combined three types of stimuli (verbal, tactile and visual) and three modes of response (verbal, pointing on one’s hand, pointing on a diagram of a hand). This brought to light on the crucial role of vision in performance and led him to conclude that finger agnosia was not due to verbal comprehension or production impairment, nor to impairment in proprioception or touch sensitivity. In contrast with our patient, Mayer reported that errors associated with Gerstmann’s syndrome affected both hands, and that finger agnosia was also present for the toes. Another difference was the absence of errors affecting the little finger, which was in stark contrast with Mr. I’s performance. A series of 26 cases reported by Basagni et al. (2021) highlighted the variability of Gerstmann’s syndrome. Despite the presence of finger agnosia and the predominant role of visual input in body representation processes, the clinical presentation of Mr. I appeared as a variant of Gerstmann’s syndrome.

Body representation requires the integration of numerous inputs (tactile, proprioceptive, visual, vestibular, nociceptive, motor, cognitive, and social, Moseley et al., 2012; Barrett, 2017; Riva et al., 2018; Havé et al., 2021; Matamala-Gomez et al., 2021). Tactile autotopagnosia may be responsible for an altered subjective personal frame that would impact multisensory integration, leading to a modified body representation. After a parietal lesion, tactile autotopagnosia may then lead to modifications in body representation. In Mr. I’s case, altered tactile perception may have led to the reweighting of the various sensory inputs, with more weight attributed to visual inputs. A greater reliance on, and confidence in, visual inputs is an adaptive strategy that could cause an efficiency gain: overestimation of the width of the hand could lead to a better discrimination of the fingers and more precise fine motor skills (Longo et al., 2015).

It is known that body ownership depends on the spatial and temporal congruence between multisensory afferents (Botvinick and Cohen, 1998; Kilteni et al., 2015). Consequently, alterations in the convergence of multisensory neural signals in the fronto-temporal-parietal junction impairs body ownership (Pazzaglia et al., 2020). In Mr. I’s case, we observed autotopagnosia in the presence of incongruent visual input. We hypothesize that the feeling of having three hands perceived in three different modalities resulted from a dysfunction in multisensory integration. The patient was disturbed by vision of the examiner’s hand moving, but also reported that under certain circumstances he could experience complete ownership of the examiner’s hand. Our paradigm allowed to replicate in laboratory settings Mr. I’s daily life experiences regarding his feeling of ownership on his son’s arm. Amazingly, we observed that during Experiment 3, when the examiner’s hand was superimposed on the position of MR. I’s hand, he had difficulty speaking and expressing himself clearly. The feeling of ownership for the examiner’s hand was so strong that Mr. I even complained about physical sensations in his right shoulder, as if his own upper limb was lying on the table in an uncomfortable position. Future work will allow more in depth investigation of the link between tactile autotopagnosia and ownership and agency experiences in Mr. I.

To conclude, this study demonstrates repercussions of difficulties in tactile perception on upper limb representation. In this clinical examination, the difficulties in localizing tactile stimuli on the right upper limb referred to altered autotopagnosia abilities. The strategy adopted by the patient reflects a strong reliance on visual afferences, but despite this he still experienced difficulties distinguishing between fingers, and a biased ownership feeling when looking at a hand that was not his own. The clinical management of patients with neurological injuries that lead to abnormal body representation can be difficult, not least because of the challenge involved in figuring out exactly what patients feel and how they perceive their body. One way in which this could be made easier, and feasible within a medical consultation, could be to design visual supports that facilitate communication about unusual bodily experiences, and help patients elaborate models as close as possible to their own experience. Such a strategy could be useful in clinical practice by providing tools for diagnosis and follow-up clinical examinations.

## Data availability statement

The raw data supporting the conclusions of this article will be made available by the authors, without undue reservation.

## Ethics statement

Written informed consent was obtained from the individual(s) for the publication of any potentially identifiable images or data included in this article.

## Author contributions

LH, FQ, EC, YR, PR, and LD contributed to conception and design of the study. LH, FQ, and A-EP organized the database and wrote the first draft of the manuscript. LH, FQ, VC, and EC performed the statistical analysis. KR, YR, and NO wrote sections of the manuscript. All authors contributed to manuscript revision, read, and approved the submitted version.

## Annex 1

### *Post hoc* comparisons for the mean x distance error for each stimulated location (Bonferroni correction)

**Table:**

		Mean difference	*t*	*p*-value
Ring finger	Little finger	153.058	4.297	0.003
	Index finger	−229.575	−6.444	<0.001
	Thumb	−303.550	−8.521	< 0.001
	Hand	−168.808	−4.739	<0.001
	Middle finger	−111.633	−3.134	0.058
Little finger	Index finger	−382.633	−10.741	<0.001
	Thumb	−456.608	−12.818	<0.001
	Hand	−321.867	−9.035	<0.001
	Middle finger	−264.692	−7.430	<0.001
Index finger	Thumb	−73.975	−2.077	0.698
	Hand	60.767	1.706	1.000
	Middle finger	117.942	3.311	0.036
Thumb	Hand	134.742	3.782	0.010
	Middle finger	191.917	5.387	<0.001
Hand	Middle finger	57.175	1.605	1.000

### *Post hoc* comparisons for the mean y distance error for each stimulated location (Bonferroni correction)

**Table:**

		Mean difference	*t*	*p*-value
Ring finger	Little finger	−48.067	−0.886	1.000
	Index finger	−11.567	−0.213	1.000
	Thumb	−174.083	−3.210	0.047
	Hand	−242.333	−4.468	0.002
	Middle finger	8.183	0.151	1.000
Little finger	Index finger	36.500	0.673	1.000
	Thumb	−126.017	−2.324	0.407
	Hand	−194.267	−3.582	0.018
	Middle finger	56.250	1.037	1.000
Index finger	Thumb	−162.517	−2.997	0.082
	Hand	−230.767	−4.255	0.003
	Middle finger	19.750	0.364	1.000
Thumb	Hand	−68.250	−1.258	1.000
	Middle finger	182.267	3.361	0.032
Hand	Middle finger	250.517	4.619	0.001
